# Comparative Analysis of Descemet Membrane Endothelial Keratoplasty (DMEK) Versus Descemetorhexis Without Keratoplasty (DSO) in Patients with Fuchs Endothelial Corneal Dystrophy

**DOI:** 10.3390/jcm14144857

**Published:** 2025-07-09

**Authors:** Vanesa Díaz-Mesa, Álvaro Sánchez-Ventosa, Timoteo González-Cruces, Alberto Membrillo, Marta Villalba-González, Alberto Villarrubia, Antonio Cano-Ortiz

**Affiliations:** 1 Department of Ophthalmology, Hospital Arruzafa, 14012 Córdoba, Spainb72depoa@uco.es (A.M.); alvillarrubia@yahoo.com (A.V.); antoniocanoortiz@gmail.com (A.C.-O.); 2 Area of Didactics of Experimental Sciences, Department of Specific Didactics, Faculty of Education and Psychology, University of Córdoba, 14071 Córdoba, Spain; 3 Department of Health and Biomedical Sciences, Loyola University Andalucía, 41704 Sevilla, Spain; 4 Faculty of Biomedical Sciences and Sports, European University of Andalucía, 29010 Málaga, Spain

**Keywords:** DMEK, DSO, cornea, fuchs dystrophy, descemetorhexis

## Abstract

**Background/Objectives**: This retrospective observational study evaluates the efficacy of Descemetorhexis without Keratoplasty (DSO) compared to Descemet Membrane Endothelial Keratoplasty (DMEK) in the management of Fuchs Endothelial Corneal Dystrophy (FECD). The outcomes were compared in terms of the corneal anatomical changes, visual results, and complication rates between the two surgical techniques for FECD. **Methods**: We conducted a retrospective, descriptive, observational study including 31 eyes from 26 patients who underwent either DSO (*n* = 16) or DMEK (*n* = 15) at the Department of Ophthalmology, Hospital Arruzafa. Patients were included if they had complete follow-up data at baseline, 6 months, and 1 year post-intervention. Their clinical information was collected from medical records and complementary tests, including the Snellen visual acuity test, Pentacam corneal tomography, and specular microscopy. **Results**: The average time to achieve best corrected distance visual acuity (CDVA) was significantly longer for DSO (7.44 ± 2.3 months) than for DMEK (5.73 ± 1.9 months, *p* = 0.004). Complication rates were higher in the DMEK group (26.7%), and in comparison, there was an absence of complications in the DSO group (*p* = 0.043). Corneal endothelial cell migration was confirmed in patients who underwent DSO, with a mean cell density of 817.17 ± 91.7 cells/mm^2^ after one year. **Conclusions**: DSO effectively treated the selected patients with FECD who presented central guttata and corneal edema, achieving visual outcomes equivalent to those of DMEK while reducing complication rates. This technique eliminates the need for donor tissue and immunosuppressive medications, making it a viable alternative for specific cases.

## 1. Introduction

The cornea is a transparent, avascular structure that constitutes the anterior part of the eyeball [1]. Its refractive power is 40 diopters, making it the main lens in the optical system. Any deformity or loss of transparency causes incorrect image projection on the retina, resulting in decreased visual acuity [2]. It has a thickness of 550 µm and consists of six layers. The external surface of the cornea is an aspherical convex shape formed by a non-keratinized stratified epithelium with defensive and regenerative properties [3]. Epithelial cells rest on Bowman’s membrane, an acellular layer about 12 µm thick composed of collagen fibrils and proteoglycans synthesized and secreted by adjacent stromal keratocytes, which provide biomechanical strength [4].

A single layer of polygonal endothelial cells about 5 µm thick lines the inner corneal surface, bordered anteriorly by Descemet’s membrane and posteriorly by the aqueous humor [3]. This layer, called the endothelium, regulates corneal homeostasis by maintaining deturgescence, essential for transparency [4]. As fluid is transported toward the stroma with nutrients, endothelial cells act as a passive barrier to prevent excessive water and solute inflow, pumping them into the anterior chamber through bicarbonate and sodium–potassium pumps [4,5].

The most common endothelial dystrophy is Fuchs Endothelial Corneal Dystrophy (FECD), affecting approximately 4% of residents over 40 years old [6]. This condition is characterized by a progressive decrease in endothelial cells, changes in the size and shape of the remaining cells, and the formation of guttata or excrescences on the Descemet membrane. These changes lead to reduced visual acuity and photophobia, making FECD the leading indication for corneal transplantation worldwide [5]. FECD involves progressive endothelial cell loss, Descemet membrane thickening, light scattering due to guttata proliferation, and progressive corneal edema, all contributing to visual impairment [7,8,9].

Historically, penetrating keratoplasty was the primary surgical treatment. However, the advent of endothelial keratoplasty techniques, particularly DMEK, has revolutionized FECD management by offering better visual outcomes and faster recovery. While DMEK provides excellent results, it also presents limitations: limited tissue availability, high cost, the potential for rejection or primary graft failure, a risk of intraocular pressure elevation due to corticosteroid use, and the possibility of rebubbling to reposition detached grafts [10].

These limitations have sparked interest in alternative approaches such as Descemet Stripping Only (DSO), which avoids the need for donor tissue and leverages the regenerative capacity of the peripheral endothelium [11]. DSO is a relatively recently developed treatment for select FECD cases. In this technique, a central area of the Descemet membrane with confluent guttata is removed, allowing healthy peripheral endothelial cells to migrate centrally and repopulate the denuded area [12,13]. For success, patient selection is critical, requiring a relatively healthy peripheral endothelium. While some studies suggest a minimum of 1000 cells/mm^2^, this number is not conclusive [14,15]. The ideal diameter for descemetorhexis is 4–5 mm, as larger areas have shown a lower probability of corneal clearing [1,16]. A major advantage of DSO is its absence of donor tissue, eliminating prolonged steroid use and reducing the risk of steroid-induced glaucoma or cataract. The main disadvantage compared to DMEK is its longer time to full corneal clearing. ROCK inhibitors (Rho kinase inhibitors), such as ripasudil 0.4% (Glanatec), have been shown to accelerate corneal clearing after DSO [3], but they were not used in this case.

On the other hand, previous research has shown that any intervention involving the corneal endothelium leads to changes in both corneal topography and pachymetry. These alterations are not limited to the posterior surface (where techniques such as DMEK or DSO are performed) but also affect the anterior surface of the cornea. These changes, which accompany the restoration of endothelial function, are essential for understanding the clinical behavior of both surgical techniques in the short and long term, as well as for explaining the functional recovery of treated patients.

Recently, numerous studies have been conducted focusing on the surgical management of FECD. However, most of these investigations lack a general consensus and do not consider specific standardization according to the various phenotypes of the disease. This study compares DSO and DMEK in the management of FECD, with a focus on corneal anatomical changes, visual recovery, and complications.

## 2. Materials and Methods

Study Design and Participants: This retrospective study included 31 eyes from 26 patients treated for FECD at Hospital Arruzafa between January 2020 and December 2022. The patients underwent either DSO (*n* = 16) or DMEK (*n* = 15). The inclusion criteria included the presence of central corneal guttata with a relatively healthy peripheral endothelium. Exclusion criteria included the presence of other ocular or systemic diseases (e.g., retinal diseases, infections, and eyelid or orbital abnormalities), previous eye surgery, choroidal hemorrhage, microphthalmos, diabetic retinopathy, pregnancy or lactation, uveitis, macular degeneration, advanced glaucoma, amblyopia, or participation in other clinical trials.

All patients received standard postoperative topical treatment consisting of dexamethasone (0.1% dexamethasone phosphate) and moxifloxacin (0.5% moxifloxacin hydrochloride) eye drops, each administered four times daily during the first postoperative week. After this period, no further corticosteroids, antibiotics, or adjunctive pharmacologic treatments were prescribed. In particular, Rho kinase inhibitors or other experimental agents were not used in any case. The use of artificial tears was allowed on an as-needed basis to alleviate symptoms of ocular surface discomfort. No additional medications or interventions were employed beyond the first postoperative week.

Surgical Techniques:

- DMEK: Removal of Descemet membrane and central endothelium (8 mm diameter), followed by transplantation of donor Descemet membrane with endothelial cells.
- DSO: Removal of central 4–5 mm of Descemet membrane without implantation of donor tissue.

Data Collection: Pre- and postoperative assessments included LogMAR visual acuity; minimum corneal thickness (µm); anterior and posterior K1, K2, and Km (µm); anterior and posterior corneal elevation (µm); anterior and posterior asphericity (µm); and spherical aberration (µm), using Pentacam imaging. Endothelial cell density (cells/mm^2^) was measured with specular microscopy reviewing the central endothelial cell density. Assessments were conducted at baseline, 6 months, and 12 months postoperatively.

Additionally, the time to achieve best visual acuity was recorded, as well as the presence of cystoid macular edema and any complications, defined as events requiring treatment or intervention. These included the reinjection of air (rebubbling) to reattach a detached graft or increases in intraocular pressure.

Statistical Analysis: Descriptive statistics (mean, standard deviation, minimum, maximum, and 95% confidence intervals) were calculated. Comparative analysis was performed using Mann–Whitney U tests for independent variables and Friedman tests for paired data, followed by Wilcoxon pairwise tests for significant results. Spearman’s correlation was used to assess the relationship between preoperative corneal thickness and the time to best visual acuity due to the assumed non-normality of the variables. All statistical tests were two-sided, with significance set at *p* < 0.05. Analyses were conducted using SPSS v.28.

## 3. Results

This comparative study between DSO and DMEK revealed notable findings across several domains. Regarding baseline characteristics, the DSO group, which included 16 eyes, comprised patients with a mean age of 61.44 years, while the DMEK group, with 15 eyes, had slightly older participants, with a mean age of 63.67 years, and there were no statistically significant differences.

The mean preoperative corrected distance visual acuity (CDVA) was 0.28 ± 0.141 LogMAR diopters for the DSO group and 0.327 ± 0.236 LogMAR diopters for the DMEK group. The mean preoperative minimum corneal thickness was 587 ± 22.09 µm for DSO and 580.07 ± 32.6 µm for DMEK. Prior to surgery, no significant differences were found between the groups in terms of CDVA or corneal thickness, ensuring a comparable baseline for analysis.

The mean preoperative anterior K1, K2, and Km values were 43.28 ± 1.56 µm, 44.28 ± 1.56 µm, and 43.75 ± 1.54 µm for DSO, and 42.46 ± 1.72 µm, 44.28 ± 1.41 µm, and 43 ± 1.62 µm for DMEK, respectively. The mean preoperative posterior K1, K2, and Km values were −5.45 ± 0.43 µm, −5.75 ± 0.38 µm, and −5.612 ± 0.4 µm in DSO, and −5.6 ± 3.44 µm, −5.81 ± 0.44 µm, and −5.8 ± 0.49 µm in DMEK.

The mean preoperative anterior corneal elevation was 0.56 ± 3.44 µm in DSO and −1 ± 5.26 µm in DMEK, while the mean posterior corneal elevation was 24.94 ± 18.72 µm in DSO and 16.5 ± 14.27 µm in DMEK. The mean preoperative anterior and posterior asphericity was −0.13 ± 0.193 µm and 0.72 ± 0.371 µm in DSO, and −0.13 ± 0.18 µm and 0.37 ± 0.82 µm in DMEK, respectively.

The mean preoperative spherical aberration was 0.16 ± 0.2 µm for the DSO group and 0.29 ± 0.19 µm for the DMEK group. The mean horizontal coma was 0.14 ± 0.23 µm in DSO and 0.029 ± 0.8 µm in DMEK, whereas the mean vertical coma was −0.08 ± 0.47 µm in DSO and −0.349 ± 0.79 µm in DMEK. Finally, the average value of total higher-order aberrations was 0.71 ± 0.25 µm in DSO and 1.25 ± 0.82 µm in DMEK. No statistically significant differences were found between these two patient populations in the preoperative evaluation.

Six months after the surgical intervention, patients who underwent DSO achieved a mean corneal thickness of 535.31 ± 28.99 µm, compared to 493.4 ± 33.88 µm in the DMEK group (*p* = 0.004). The mean posterior corneal elevation was 20.19 ± 15.34 µm for DSO versus 7 ± 4.13 µm for DMEK (*p* = 0.003). Posterior asphericity in the DMEK group was greater (−0.216 ± 0.43 µm) than in the DSO group (−0.6 ± 0.29 µm), with a significance of 0.023. The remaining variables did not show statistically significant results (Table 1).

These trends remained similar one year after surgery. The DSO group (535.31 ± 28.99 µm) showed a greater corneal thickness than the DMEK group (493.4 ± 33.88 µm), with a significance of 0.004. The mean posterior K2 value for DSO was −6.63 ± 0.25 µm, in contrast with −6.36 ± 0.32 µm for DMEK (*p* = 0.038). The mean posterior asphericity remained higher in the DSO group (−0.57 ± 0.4 µm) compared to the DMEK group (−0.26 ± 0.16 µm) (*p* = 0.011). Table 2 and Table 3 show the progression of the preoperative and one-year data.

**Figure 1:**
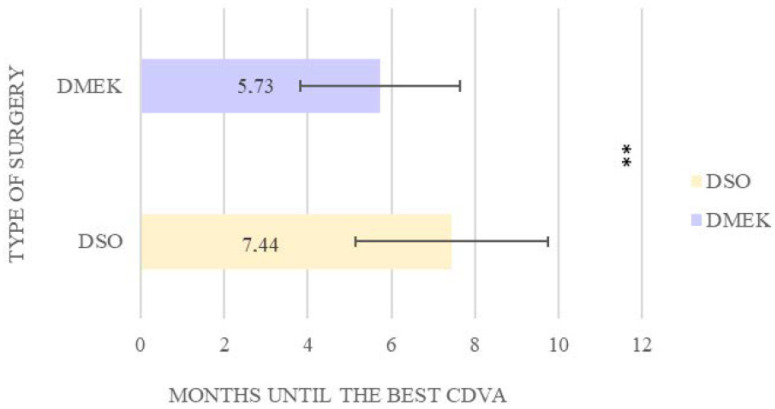
Time to achieve the best CDVA between DMEK and DSO. ** Statistically significant differences.

Visual recovery during the one-year follow-up showed significant improvement in both groups. CDVA reached comparable levels of 0.07 LogMAR in the DSO group and 0.02 LogMAR in the DMEK group (*p* > 0.05). However, the time required to achieve this improvement differed significantly. Patients in the DSO group required a longer recovery period, with an average of 7.44 months to reach their optimal CDVA, compared to 5.73 months in the DMEK group (*p* = 0.004). Although the younger patients in both groups tended to recover slightly faster, this trend did not reach statistical significance (*p* > 0.05) (Figure 1).

**Figure 2:**
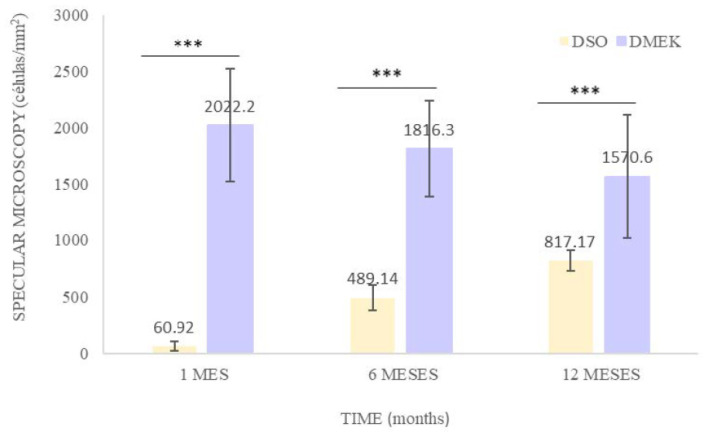
Cell count per mm^2^ in DMEK vs. DSO. *** Statistically significant differences.

The mean endothelial cell count per square millimeter at one month, six months, and one year was 60.92 ± 39.46 and 817.17 ± 91.7 in the DSO group, and 2022.2 ± 498.26, 1816.3 ± 420.48, and 1570.6 ± 548.35 in the DMEK group (*p* < 0.001) (Figure 2).

In our DMEK group, four patients experienced complications (26.7%), including elevated intraocular pressure (*n* = 2) and the need for rebubbling (*n* = 2). However, no complications were reported in the DSO group (*p* = 0.043). Neither group developed cystoid macular edema.

Complications represented another critical area of comparison. The DMEK group had a higher incidence of postoperative complications, with 26.7% (*n* = 4) of patients experiencing issues such as elevated intraocular pressure (*n* = 2) and the need for rebubbling (*n* = 2) to reposition the graft. In contrast, the DSO group reported no complications during the study period. Neither group developed cystoid macular edema. All four patients eventually recovered successfully.

## 4. Discussion

This study demonstrates the efficacy of DSO as a tissue-preserving technique, providing a compelling alternative for the management of central FECD. Although patients treated with DSO required longer recovery times to achieve optimal visual outcomes, these results were ultimately comparable to those of DMEK. This finding is consistent with previous research highlighting the significant regenerative capacity of peripheral endothelial cells [11].

A gradual improvement in visual acuity may occur many months after surgery in both corneal transplant patients and those undergoing DSO. For this reason, visual acuity was assessed at six months and one year after surgery, thus establishing a clear evaluation criterion. In line with Huang MJ et al., we demonstrated that DSO (CDVA of 0.09 ± 0.127 LogMAR and 0.034 ± 0.92 LogMAR at six months and one year, respectively) provides final visual outcomes equivalent to DMEK (CDVA of 0.06 ± 0.116 LogMAR and 0.021 ± 0.028 LogMAR at six months and one year, respectively), while significantly reducing the complication rate. All eyes showed a clinically apparent improvement in central corneal edema, along with a subjective improvement in vision [16]. In contrast to their study, ours featured an equal follow-up time in both groups, eliminating the possibility of bias in the results.

Regarding corneal parameters, the DSO-treated patient group (539.69 ± 29 µm) exhibited a higher mean minimum corneal thickness than DMEK (488.09 ± 43.92 µm) six months after treatment, likely related to the longer time required for corneal clearing in DSO. For this same reason, the mean posterior corneal elevation remained higher in DSO (20.19 ± 15.34 µm) than in DMEK (7 ± 4.13 µm). However, posterior asphericity was greater in DMEK (−0.216 ± 0.43 µm) than in DSO (−0.6 ± 0.29 µm). One year after surgery, this increase in DSO compared to DMEK was again observed. However, the K2 value was higher for DMEK (−6.36 ± 0.32) than for DSO (−6.63 ± 0.25). The corneal clearing capacity or “pumping strength” appeared to be greater in DMEK than in DSO.

Paired statistical analysis confirmed that the number of endothelial cells per square millimeter in the central corneal endothelium that migrated to the damaged central corneal tissue was higher at one year (817.17 ± 91.7) compared to six months (489.14 ± 112.55) and one month (60.92 ± 39.46) (*p* = 0.002). In contrast, the DMEK group maintained a relatively stable endothelial cell count throughout the follow-up period, with a mean density of 1570.6 cells/mm^2^ after one year, reflecting the preservation of transplanted donor cells. This progress highlights the migratory potential of the peripheral endothelium. Evidence of endothelial cell migration and regeneration in eyes treated with DSO aligns with emerging data suggesting that the peripheral endothelium can effectively repopulate damaged areas. This process not only restores corneal clarity but also reinforces the concept of endothelial plasticity as a critical factor in the management of FECD [17].

Corneal endothelial cells (CECs) form a hexagonal monolayer on the posterior surface of the cornea and play an essential role in maintaining corneal transparency by actively regulating fluid balance through a basolaterally located NA^+^/K^+^- ATPase pump system [18]. Unlike other cell types, human CECs exhibit an extremely limited proliferative capacity in vivo, which prevents effective regeneration after injury. As a result, endothelial cell density declines progressively with age—from approximately 4000 cells/mm^2^ in early life to fewer than 2000 cells/mm^2^ in older adults—compensated only by cellular enlargement and morphological changes such as polymegathism and pleomorphism [19]. This gradual physiological deterioration predisposes the cornea to endothelial dysfunction, stromal edema, and, ultimately, vision loss, particularly following intraocular surgery or in the context of diseases such as Fuchs Endothelial Corneal Dystrophy.

From a clinical perspective, this structural vulnerability has driven the development of more selective and less invasive therapeutic strategies. Procedures such as DMEK and DSO, along with recent advances in cell-based therapies—including ex vivo CEC culture, intracameral cell injection, and pharmacologic approaches using ROCK inhibitors—offer promising alternatives for restoring endothelial function without the need for full-thickness corneal transplantation [20]. A deeper understanding of corneal endothelial physiology not only contextualizes these innovative approaches but also helps interpret clinical outcomes and optimize therapeutic decision-making based on the functional status of the endothelium.

On average, patients treated with DSO took 7.44 ± 2.3 months to reach their best visual acuity, compared to 5.73 ± 1.9 months in the DMEK group. DSO presents a significantly longer corneal clearing time compared to DMEK, representing its main drawback. However, the reduction in complication rates observed with DSO supports its utility as a safer option, particularly for patients who may be at higher risk for graft-related complications associated with DMEK (26.7% in our case). For example, DSO patients avoided common DMEK complications such as elevated intraocular pressure (by eliminating the need for immunosuppressive eye drops) and rebubbling, underscoring the potential of this technique to minimize postoperative interventions [16].

Moreover, the lower reliance on donor tissue positions DSO as a cost-effective and resource-conscious option in corneal surgery. This advantage addresses a significant limitation of DMEK, which depends on donor tissue availability and carries inherent risks associated with long-term immunosuppression [21].

Recent discussions have questioned whether DSO could serve as a safe and effective alternative to traditional endothelial transplantation in patients with early-stage FECD. By avoiding donor tissue, DSO eliminates the risk of immune rejection, making it an attractive option for selected patient profiles. Nevertheless, DMEK remains the gold standard in more advanced cases of endothelial dysfunction due to its superior visual outcomes, minimal rejection rates, and faster postoperative recovery. The choice between these approaches should be based on endothelial cell status, morphology, and individual surgical risk. These advances highlight the relevance of a comprehensive understanding of endothelial physiology and support the need to tailor therapy based on functional tissue characteristics—an approach directly aligned with the objectives of the present study [22]. Nevertheless, these findings should be interpreted within the limitations of this study, including its retrospective design and relatively small sample size. Larger prospective trials are needed to validate these results and establish clear patient selection guidelines [17]. Furthermore, future research should investigate the long-term durability of endothelial regeneration in DSO and its implications for advanced cases of FECD.

In this context, the ongoing DETECT II trial, a multicenter, randomized, assessor-masked study comparing DMEK and DSO combined with the Rho kinase inhibitor ripasudil represents a pivotal step toward clarifying the clinical efficacy and regenerative potential of DSO-based therapies. Its outcomes may help define evidence-based indications for less invasive approaches in early to moderate FECD [23].

## 5. Conclusions

DSO is a viable alternative to DMEK for patients with FECD who present specific phenotypes. This technique achieves visual outcomes equivalent to those of DMEK, demonstrating its ability to effectively restore visual acuity. The absence of complications such as graft detachment or elevated intraocular pressure highlights its safety profile, making it particularly advantageous for patients with higher surgical risk.

Moreover, its reduced reliance on donor tissue and immunosuppressive medications positions DSO as a cost-effective and resource-conscious option in corneal surgery.

This study represents the first direct comparison of such a wide range of corneal parameters between the two techniques, yielding statistically significant differences. In addition, the migratory capacity of endothelial cells toward areas of damage on the cornea was confirmed.

In conclusion, DSO represents a paradigm shift in the management of FECD, particularly for cases in which resource limitations or patient-specific risks prevent traditional DMEK. Its potential to reduce healthcare costs while maintaining excellent clinical outcomes makes it an essential addition to the repertoire of surgical options for corneal diseases. Continued innovation and larger-scale studies are essential to fully integrate DSO into widespread clinical practice.

## Figures and Tables

**Table 1:** Quantitative bivariate analysis at 6 months.

	Mean ± DS		MIN		MAX		*p*
	DSO	DMEK	DSO	DMEK	DSO	DMEK	
CDVA	0.09 ± 0.13	0.06 ± 0.16	0.301	0.699	−0.079	−0.079	0.275
Thinnest pachymetry	539.69 ± 29.00	488.09 ± 43.92	491	437	580	572	0.006 *
Anterior K1	42.98 ± 1.64	41.83 ± 1.63	41	39.3	47.3	43.6	0.226
Anterior K2	44.05 ± 1.64	42.92 ± 1.40	42.2	41.1	48.4	44.6	0.054
Anterior Km	43.49 ± 1.59	42.54 ± 1.49	41.8	40.2	47.8	44.1	0.225
Posterior K1	−6.318 ± 0.39	−6.25 ± 0.27	−7.1	−6.6	−5.6	−5.6	0.71
Posterior K2	−5.82 ± 3.40	−6.57 ± 0.33	−7.2	−7	6.9	−5.8	0.791
Posterior Km	−6.50 ± 0.31	−6.41 ± 0.29	−7.1	−6.7	−6	−5.7	0.576
Anterior Elevation	−1.56 ± 4.84	−0.60 ± 4.81	−15	−8	5	10	0.915
Posterior Elevation	20.19 ± 15.34	7.00 ± 4.14	5	2	58	15	0.003 *
Anterior Q	−0.136 ± 0.243	−0.12 ± 0.36	−0.49	−0.69	0.36	0.69	0.863
Posterior Q	−0.60 ± 0.43	−0.21 ± 0.29	−1.46	−0.64	0.15	0.25	0.023 *
Spherical aberration	0.416 ± 0.186	0.469 ± 0.259	0.118	0.099	0.721	0.913	0.616
Horizontal coma	0.209 ± 0.260	0.007 ± 0.456	−0.29	−0.62	0.561	0.716	0.155
Vertical coma	0.315 ± 0.548	0.209 ± 0.383	−0.322	−0.38	1.687	1.064	0.895
HOAS	0.897 ± 0.403	0.942 ± 0.411	0.407	0.485	1.877	1.796	0.712

CDVA: corrected distance visual acuity; K1: flat corneal meridian; K2: steep corneal meridian; HOAS: Higher-Order Aberrations; * Statistically significant differences.

**Table 2:** Quantitative bivariate analysis at the preoperative stage.

	Mean ± DS		MIN		MAX		*p*
	DSO	DMEK	DSO	DMEK	DSO	DMEK	
CDVA	0.28 ± 0.14	0.33 ± 0.24	0.824	0.824	0.125	0	0.182
Thinnest pachymetry	587.00 ± 22.09	580.07 ± 32.60	536	526	616	634	0.464
Anterior K1	43.28 ± 1.57	42.47 ± 1.72	40.9	39.5	46.8	44.8	0.451
Anterior K2	44.29 ± 1.57	43.61 ± 1.42	42	40.9	47.7	44.9	0.429
Anterior Km	43.78 ± 1.54	43.00 ± 1.62	41.7	40.4	47.2	44.8	0.506
Posterior K1	−5.46 ± 0.44	−5.6 ± 0.55	−6.3	−6.3	−4.9	−4.7	0.382
Posterior K2	−5.75 ± 0.39	−5.81 ± 0.44	−6.4	−6.6	−5.3	−5	0.504
Posterior Km	−5.61 ± 0.40	−5.81 ± 0.47	−6.3	−6.5	−5.2	−5.1	0.26
Anterior Elevation	0.56 ± 3.44	−1 ± 5.26	−6	−15	6	6	0.785
Posterior Elevation	24.94 ± 18.72	16.5 ± 14.271	−1	−1	66	43	0.22
Anterior Q	−0.134 ± 0.193	−0.126 ± 0.179	−0.38	−0.6	0.26	0.15	0.54
Posterior Q	0.723 ± 0.501	0.371 ± 0.822	0	−0.55	1.82	1.7	0.17
Spherical aberration	0.158 ± 0.204	0.296 ± 0.189	−0.479	−0.07	0.428	0.574	0.084
Horizontal coma	0.141 ± 0.233	0.029 ± 0.802	−0.15	−2.15	0.614	1.092	0.901
Vertical coma	−0.08 ± 0.473	−0.349 ± 0.795	−0.83	−1.63	0.803	0.757	0.647
HOAS	0.711 ± 0.258	1.253 ± 0.822	0.294	0.381	1.256	2.693	0.17

CDVA: corrected distance visual acuity; K1: flat corneal meridian; K2: steep corneal meridian; HOAS: Higher-Order Aberrations.

**Table 3:** Quantitative bivariate analysis one year after.

	Mean ± DS		MIN		MAX		*p*
	DSO	DMEK	DSO	DMEK	DSO	DMEK	
CDVA	0.034 ± 0.092	0.021 ± 0.028	0.204	0.070	−0.09	0	0.572
Thinnest pachymetry	535.31 ± 28.99	493.4 ± 33.88	485	455	583	570	0.004 *
Anterior K1	42.83 ± 1.61	41.74 ± 1.74	41	39.1	47.2	44.1	0.257
Anterior K2	43.81 ± 1.63	42.94 ± 1.55	41.6	40.7	48.4	44.6	0.279
Anterior Km	43.31 ± 1.62	42.33 ± 1.64	41.6	40	47.8	44.3	0.225
Posterior K1	−6.26 ± 0.31	−6.1 ± 0.34	−6.8	−6.5	−5.7	−5.6	0.325
Posterior K2	−6.63 ± 0.25	−6.36 ± 0.32	−6.9	−6.9	−6.2	−5.8	0.038 *
Posterior Km	−6.44 ± 0.26	−6.22 ± 0.33	−6.9	−6.7	−6	−5.7	0.131
Anterior Elevation	−1 ± 3.578	−0.7 ± 4.692	−8	−9	6	6	0.77
Posterior Elevation	18.31 ± 15.899	9 ± 6.549	3	0	63	22	0.086
Anterior Q	−0.053 ± 0.273	−0.194 ± 0.224	−0.43	−0.7	0.61	0.03	0.292
Posterior Q	−0.577 ± 0.408	−0.261 ± 0.158	−1.24	−0.68	0.12	−0.11	0.011 *
Spherical aberration	0.435 ± 0.219	0.299 ± 0.129	0.167	0.069	1.003	0.455	0.206
Horizontal coma	0.14 ± 0.18	0.136 ± 0.346	−0.229	−0.43	0.361	0.663	0.732
Vertical coma	0.329 ± 0.453	0.229 ± 0.477	−0.408	−0.25	1.166	1.036	0.343
HOAS	0.916 ± 0.565	0.848 ± 0.319	0.415	0.486	2.776	1.251	0.916

CDVA: corrected distance visual acuity; K1: flat corneal meridian; K2: steep corneal meridian; HOAS: Higher-Order Aberrations; * Statistically significant differences.

## Data Availability

The anonymized data from this study may be made available upon reasonable request to the corresponding author, subject to a formal request and a justified purpose.

## Abbreviations

The following abbreviations are used in this manuscript:

FECD	Fuchs Endothelial Corneal Dystrophy
DMEK	Descemet Membrane Endothelial Keratoplasty
DSO	Descemet Stripping Only
CDVA	Corrected Distance Visual Acuity
